# Neurological prognostication after cardiac arrest: evidence review and institutional protocol proposal

**DOI:** 10.1055/s-0046-1829052

**Published:** 2026-10-02

**Authors:** Eduardo Drews Amorim, Rose Plotnik, Raphael Machado Castilhos

**Affiliations:** 1 Hospital de Clínicas de Porto Alegre, Serviço de Neurologia, Porto Alegre RS, Brazil.; 2 Universidade Federal do Rio Grande do Sul, Programa de Pós-Graduação em Ciências Médicas, Porto Alegre RS, Brazil.; 3 Hospital de Clínicas de Porto Alegre, Serviço de Medicina Intensiva, Porto Alegre RS, Brazil.; 4 Universidade Federal do Rio Grande do Sul, Departamento de Medicina Interna, Porto Alegre RS, Brazil.

**Keywords:** Heart Arrest, Prognosis, Hypoxia-Ischemia, Brain, Clinical Protocols

## Abstract

**Background:**

Cardiac arrest (CA) is associated with high mortality and neurological morbidity, mainly due to hypoxic-ischemic encephalopathy. Accurate neurological prognostication in CA survivors is essential to guide therapeutic decisions and provide appropriate care.

**Objective:**

To review the current evidence on neurological prognostication after CA and to propose an institutional protocol adapted to the resources of Hospital de Clínicas de Porto Alegre (HCPA).

**Methods:**

The current narrative review was conducted in two stages: a literature review and the development of an institutional protocol. Articles were selected according to their clinical relevance and their contribution to current evidence on neurological prognostication after CA. The PubMed, Scopus, Web of Science, Embase, and SciELO databases were searched. The protocol was developed considering the clinical tools available at HCPA, including neurological examination, electroencephalography (EEG), and neuroimaging.

**Results:**

Brain injury after CA occurs in three stages: intra-arrest ischemia, immediate reperfusion, and delayed postreperfusion injury. Prognostic modalities include neurological examination, EEG, somatosensory-evoked potentials (SSEPs), serum biomarkers, and neuroimaging. Reliable predictors of poor outcome include absent brainstem reflexes at 72 hours, persistent EEG suppression, bilateral absence of cortical N20 potentials, and diffuse abnormalities on magnetic resonance imaging. The proposed protocol organizes assessments from admission to the Intensive Care Unit through day 5 after the return of spontaneous circulation.

**Conclusion:**

A standardized post-CA neurological prognostication protocol at HCPA may improve care quality, enhance communication with families, and support ethically-sound decision-making.

## INTRODUCTION

Cardiac arrest (CA) is defined as the abrupt and unexpected cessation of spontaneous and effective ventilation and circulation. 1 It is a catastrophic event with extremely-high mortality, typically triggered by an acute or chronic severe underlying condition. When CA occurs, cerebral blood flow ceases, and consciousness is lost within seconds. If not promptly managed, irreversible brain damage and death may ensue. The likelihood of survival or of achieving a favorable neurological outcome declines rapidly as the time until the return of spontaneous circulation (ROSC) increases. It is estimated that approximately 500 thousand patients undergo cardiopulmonary resuscitation (CPR) annually during hospitalization, and an additional 225 thousand individuals receive CPR in out-of-hospital settings. 2 In the United States, only 14% of these patients survive. 3

As the heart is more tolerant to ischemia than the brain, epidemiological data indicate that nearly 70% of patients admitted to the hospital after CA die from the consequences of brain injury, even when initial resuscitation efforts are successful. 4 Postresuscitation brain injury can vary in severity, ranging from mild impairment to devastating brain injury and brainstem death. 2 Early initiation of high-quality CPR and defibrillation, when indicated, are primary goals in the initial management of patients with CA, aiming to minimize ischemic brain damage and optimize recovery chances.

Neurological injury after CA can be subdivided into three stages:

- intra-arrest ischemic injury;
- immediate reperfusion injury (beginning with delayed ROSC and lasting several hours); and
- delayed postreperfusion injury (beginning hours after ROSC, peaking at 12–24 hours, and continuing for up to 5 days). 2

Neurological prognosis following CA is highly variable and depends on multiple factors, including the cause of arrest, time until resuscitation, quality of CPR, and subsequent therapeutic interventions. Hypoxic-ischemic (HI) brain injury is the leading cause of morbidity and mortality among CA survivors, especially in out-of-hospital CA cases. 5

The best neurological outcomes are observed in patients who regain consciousness early—ideally within the first 96 hours after the event. However, most successfully-resuscitated patients present in a comatose state due to (HI) encephalopathy. 2 In many such cases, it becomes necessary to assess the likelihood of a poor neurological outcome using prognostic tools, which may guide decisions regarding the withdrawal of life-sustaining therapies, initiation of family discussions, and consideration of organ donation. Neurological prognostication in post-CA patients involves five key modalities: clinical neurological examination, neuroimaging (preferably magnetic resonance imaging, MRI), electroencephalography (EEG), somatosensory-evoked potentials (SSEPs), and specific serum biomarkers. 6 7 These tools generally have high specificity to predict poor neurological outcomes, although the sensitivity can vary. 8

The objective of the current study is to conduct a comprehensive literature review of the most recent evidence regarding neurological prognosis in CA survivors. Based on this review, an institutional protocol will be developed for Hospital de Clínicas de Porto Alegre (HCPA), tailored to the resources available at this institution, with the goal of standardizing the management and evaluation of these patients.

## METHODS

The present study will be conducted in two main stages: a literature review and the development of an institutional protocol. It adopts a qualitative approach aimed at identifying, selecting, and analyzing relevant studies on the neurological prognosis of CA survivors, with the goal of developing a protocol tailored to the context and available resources at HCPA.

For the literature review, the following scientific databases were consulted: PubMed, Scopus, Web of Science, Embase, and SciELO. The search strategy used the following keywords: *cardiac arrest* , *neurological prognosis* , *postcardiac arrest care* , and *neuroprognostication* . Articles were included if they were published in English, Spanish, or Portuguese, and consisted of original research, systematic reviews, or clinical guidelines addressing the neurological management and prognosis of CA survivors. Studies focusing on pediatric populations or those dealing exclusively with ethical or legal aspects without clinical relevance were excluded. A narrative synthesis was performed to integrate the findings.

For the development of the protocol, a document was created based on the collected evidence and adapted to the resources and infrastructure available at HCPA. The protocol structure included: identification of eligible patients, methods for initial and ongoing neurological prognostic assessment, clinical management guidelines, and flowcharts to support the multidisciplinary care team. The protocol was reviewed by experts in neurology and intensive care, and it will be submitted to the hospital's Ethics Committee for validation and revision prior to implementation.

Finally, the study strictly adhered to ethical principles and Brazilian national research standards in health, ensuring transparency at all stages. As there was no direct involvement of human subjects, and the study consisted solely of a literature review and the development of an institutional document, ethical considerations were aligned with current guidelines for this type of research.

## RESULTS

### Pathophysiology and management of consequences

The severity and duration of ischemia during CA determine the extent of primary neurological injury (no-flow), whereas secondary injury occurs during CPR (low-flow) and after ROSC (reperfusion). 2

#### *Primary injury (minutes to hours)*

Neuronal injury is rapidly triggered in the brain when the severity and duration of ischemia are sufficient to cause sustained depolarization of the neuronal plasma membrane, known as *ischemic depolarization* . This primary injury occurs within minutes after CA due to the cessation of cerebral blood flow and the subsequent depletion of oxygen, glucose, and adenosine triphosphate (ATP). The resulting equalization of ionic gradients during ischemia leads to pathological cellular edema, intracellular calcium overload, and activation of deleterious proteases. To mitigate the extent of primary injury, the most effective strategy is early and high-quality CPR, along with prompt defibrillation, aiming to achieve the shortest possible time until ROSC. 2

#### *Secondary injury (hours to days)*

After partial or complete restoration of blood flow, persistent or recurrent inadequate cerebral-oxygen delivery may lead to secondary brain injury. Contributing factors include hypoxemia, inadequate cerebral perfusion pressure (CPP) due to refractory hypotension, or elevated intracranial pressure (ICP) secondary to cerebral edema. Clinical or subclinical seizures, hyperglycemia, and hyperthermia may also increase cerebral metabolic demand, further exacerbating secondary brain injury. 2

The systemic inflammatory response in patients following CA is associated with multiorgan dysfunction and death. In addition to systemic inflammation, activation of neuronal immune and inflammatory cascades—likely detrimental to neuronal survival—occurs within hours of ischemia onset. Cerebral edema following ischemia may develop rapidly, increase ICP, and impair regional and global cerebral perfusion. Immediately after ROSC, it is recommended to avoid hypoxemia and hypotension, and to initiate targeted temperature management (TTM) in comatose patients. This involves controlled reduction of core body temperature, measured via an esophageal thermometer, to a target between 32 °C and 37.5 °C for a minimum of 36 hours, followed by gradual rewarming. 9 Additionally, fever should be strictly avoided in all post-CA patients for at least 72 hours. 10 11

Cerebral blood flow and oxygen delivery are also influenced by arterial oxygen and carbon dioxide levels; therefore, current guidelines recommend maintaining normoxia and normocapnia after ROSC. Moreover, higher mean arterial pressure (MAP) targets (80–100 mmHg) may be necessary in some patients to ensure adequate cerebral-tissue oxygenation, likely due to perivascular cellular edema or cerebral capillary collapse. 12

Seizures occur in 20 to 30% of post-CA patients in the intensive care unit (ICU), and they are generally indicative of severe brain injury. 2 These should be promptly treated with first-line antiepileptic drugs such as levetiracetam or sodium valproate, in addition to sedative agents; however, it should be noted that intravenous sodium valproate is not widely available in Brazil. 13 Hyperglycemia is common after CA and, although definitive evidence of benefit is lacking, current recommendations suggest treating hyperglycemia with continuous insulin infusion, targeting blood glucose levels between 140 and 180 mg/dL.

### Postcardiac arrest outcomes

Historically, the prognosis following CA has been poor, with a reported survival rate of approximately 14% in some studies conducted in the United States. 3 6 However, mortality rates and the incidence of severe neurological impairment have declined in recent years due to the implementation of therapeutic temperature modulation techniques, more aggressive treatment of secondary brain injury related to ischemic time, and improved care during the early stages of CA (pre- and in-hospital). 6

In summary, survival after CA depends on multiple factors: the location of the arrest (patients with in-hospital cardiac arrest have higher chances of survival compared with those experiencing out-of-hospital events), proximity to a first responder, access to defibrillation in the case of a shockable rhythm, the quality of CPR, implementation of TTM, and the quality of postresuscitation intensive care. 14 15 Clinical outcomes should be assessed at a minimum of 3 months—and ideally for 6 months or longer—following the cardiac arrest. 16

### Cognitive and functional impacts

Hypoxic-ischemic encephalopathy frequently results in cognitive and functional sequelae in patients who survive CA. In the early stages of recovery, dysfunction is typically driven by selective hippocampal injury within the first 72 hours postevent, manifesting as deficits in immediate memory. Over time, however, patients often experience impairments across a broader range of cognitive domains—most notably memory and executive function—indicating global cognitive dysfunction. 17 Cognitive improvement usually occurs within the first 3 months after the event, with minimal additional recovery beyond 6 to 12 months. 18 Furthermore, some patients may experience progressive and accelerated cognitive decline over time, resembling a neurodegenerative disorder. 19

Neurological manifestations resulting from anoxic injury vary according to the affected brain regions. Damage to the thalamus, cortex, or brainstem is often associated with alterations in consciousness and attention, including coma in severe cases. Injury to the cerebellum and basal ganglia frequently results in myoclonus, movement disorders, and motor incoordination. Hippocampal damage tends to cause memory deficits. Extensive involvement of the brainstem may lead to hemodynamic disturbances, including dysregulation of blood pressure, body temperature, heart rate, and respiratory control. Finally, cortical and thalamic lesions are associated with impairments in attention, information processing, and executive function. 20

Predictors of worse cognitive outcomes after cardiac arrest include advanced age, prolonged time until ROSC, and longer duration of hospitalization. 19 For reasons not yet fully understood, women have been found 21 to experience poorer cognitive, functional, and psychiatric outcomes following hospital discharge. However, it is important to move away from the traditional assumption that survival after CA inevitably leads to devastating neurological outcomes, as many patients can achieve meaningful cognitive recovery and return to a quality of life similar to that prior to the event.

### Goals of care

Most patients who survive the initial insult of CA ultimately die following the active withdrawal of life-sustaining therapies, based on the presumption that neurological recovery is unlikely. The duality between pessimism grounded in a grim neurological prognosis and the hope for patient awakening, even in the face of signs suggesting that such recovery is improbable, reflects the complex and controversial life-or-death decisions inherent in these situations. This difficult reality highlights the pressing need for accurate and reliable neuroprognostic tools, enabling clinicians to guide therapeutic decisions appropriately and individually, while also considering the values, wishes, and emotional perspectives of the patient's family. 6

### Neuroprognostication after cardiac arrest

There are five essential modalities that support neurological prognostication following CA: neurological examination, EEG, SSEPs, serum biomarkers, and neuroimaging. 22 When used together, these tools enable a multimodal approach to predict the outcome of HI brain injury and identify patients with a poor neurological prognosis.

#### *Neurological examination*

The clinical examination is the first step in neurological prognostication. It should be performed in the absence of therapeutic hypothermia (TH) or sedative medications, as both can suppress brain function and mask neurological responses. Moreover, brainstem reflexes may spontaneously recover within the first 48 hours after the event. Therefore, it is recommended that the initial neurological assessment be conducted at least 72 hours after CA, once sedatives have been fully cleared and confounding factors such as infections or electrolyte imbalances have been corrected. 14 Risk factors for delayed awakening from coma include renal failure, advanced age, and postarrest hemodynamic shock. 23

A complete neurological examination in a comatose patient includes the assessment of wakefulness, verbal response and command-following ability, brainstem reflexes, response to painful stimuli, and motor responses. Among these, brainstem reflexes and motor response to pain are particularly informative. In comatose patients, localization to pain is a predictor of a favorable neurological outcome, although preserved brainstem reflexes are not considered reliable indicators of favorable clinical outcome. 22 In contrast, the bilateral absence of pupillary light reflexes remains one of the strongest clinical predictors of poor neurological outcome following CA. ^22^ Although the corneal reflex has been historically cited as a prognostic marker, current guidelines emphasize that, unlike the pupillary light reflex, the corneal reflex is not a reliable predictor of neurological outcome after CA. 16

The presence of myoclonus within the first 48 hours after CA is also a key clinical feature to assess. If detected, an EEG should be performed to determine the origin of the myoclonus (cortical, subcortical, or other) and to investigate epileptic activity. However, myoclonus alone is not considered a reliable predictor of poor outcome, unless accompanied by additional unfavorable prognostic markers. Myoclonic status epilepticus (SE), on the other hand, has been traditionally associated with a poor prognosis when it occurs within the first 48 hours after the arrest. It is important to note that Lance-Adams syndrome, characterized by the late onset and persistent subcortical myoclonus in CA survivors, is often associated with good functional recovery. 16

While the neurological examination is essential to evaluate current brain function, it is not fully accurate on its own. Therefore, a multimodal approach is recommended, incorporating complementary tests to improve the precision of brain-injury assessment. 6

#### *Continuous electroencephalography monitoring*

EEG is a non-invasive, low-cost, and widely-available tool that plays an important role in post-CA neurological prognostication. 24 Detection of myoclonic SE, generalized periodic discharges (GPDs), and malignant EEG patterns—such as generalized suppression, sharp waves, polyspikes, and spike-and-wave complexes—occur in up to 40% of the patients. Early seizure activity (within 48 hours of the event) is associated with poor prognosis; therefore, continuous EEG monitoring is recommended during this critical window to increase sensitivity for seizure detection. 25 Similarly, low-voltage EEG patterns (< 10 μV) are strongly associated with unfavorable outcomes. In contrast, the evolution from a discontinuous to a continuous background pattern is a favorable prognostic sign, as is the presence of EEG reactivity to external stimuli (such as hand clapping, pinching), especially when these findings are observed within the first 24 hours after ROSC. 24

Defining benign versus malignant EEG patterns is essential for standardized assessment across patients. Background suppression is defined as a voltage < 10 μV. The EEG patterns are categorized as *continuous* , *discontinuous* , *nearly continuous* , *burst attenuation/suppression* , or *suppressed* . Most studies 26 have shown that persistent burst suppression lasting more than 24 hours almost always predicts poor neurological outcome, with a specificity close to 100%.

Although EEG activity can be affected by sedative drugs, it is only minimally influenced by therapeutic hypothermia. Nonetheless, continuous EEG monitoring within the first 24 hours after CA is recommended for seizure detection, even in patients receiving ongoing sedation. Sedative regimens that do not significantly suppress EEG activity during this period include midazolam at 0.1 to 0.2 mg/kg/h and propofol at 2 to 3 mg/kg/h. 24

### Somatosensory-evoked potentials

The SSEPs are a valuable, though not so widely available, tool for neurological prognostication in post-CA patients. A key advantage is their relative resistance to the confounding effects of continuous sedation and TH. 27 They are small electrical signals (< 10–50 μV) recorded from the scalp following peripheral stimulation, typically of the median nerve at the wrist. 28 Early-latency responses are recorded ipsilaterally at the elbow, brachial plexus, or cervical spinal cord, while the N20 cortical potential—critical for prognostication—is recorded at the contralateral postcentral gyrus. It is important to note that cortical N20 responses are only interpretable if peripheral and spinal responses are preserved. 28

The main limitations of SSEPs include issues with reproducibility and interpretability, as few centers have the necessary equipment and experienced personnel to perform and analyze the test. Additionally, SSEPs can be affected by electrical noise and interference from equipment commonly present in the intensive-care setting. 6

Despite these challenges, SSEPs are the most thoroughly-studied and validated predictor of poor neurological outcome following CA. The bilateral absence of cortical N20 responses, when assessed more than 72 hours after the event, is almost invariably associated with a poor long-term neurological prognosis. 29 30 However, although highly specific, SSEPs have a sensitivity of approximately 50%, indicating that many patients with preserved N20 responses may still experience poor neurological outcomes. 29 It is recommended 28 that SSEPs be performed after 72 hours of the event, as absent responses during the hypothermia phase may reappear during rewarming.

The SSEPs are especially helpful in identifying patients with a dismal prognosis, and they may aid families facing difficult decisions regarding goals of care. However, they are not currently reliable to predict favorable outcomes. Recent studies 30 have explored whether greater N20 amplitude may be associated with good prognosis, but evidence on this point remains limited.

#### *Serum biomarkers of neuronal injury*

Several serum biomarkers of neuronal injury can provide insight into the extent of neurological damage following CA. However, limited accessibility and lack of precision remain significant barriers to their routine use.

Neuron-specific enolase (NSE) is one of the most validated biomarkers in this context. Serum NSE levels typically peak around 24 hours after ROSC and should be measured at 24, 48, and 72 hours. In patients with poor neurological prognosis, NSE levels remain persistently elevated, and a serum level above 33 μg/L within the first 72 hours after CA is considered a threshold for poor outcome in normothermic patients. 31 In patients treated with HT, NSE levels tend to be lower, which reduces the test's predictive accuracy. 32 However, NSE concentrations exceeding 90 μg/L, despite targeted temperature management, are associated with a 99% of specificity for unfavorable neurological outcomes. 33 It is important to note that serum NSE can be affected by non-neurological conditions such as NSE-producing tumors or hemolysis. 34

Similarly, S-100β, an astroglial protein, is considered a marker of poor prognosis when found at high serum concentrations more than 72 hours after CA; however, this test is even less widely-available and requires further validation through additional studies. 35 Likewise, large-scale studies 36 have identified neurofilament light chain (NfL) as a promising biomarker for neurological prognosis after CA, with high sensitivity and specificity, although more research is needed to confirm these preliminary findings.

#### *Neuroimaging*

Brain magnetic resonance imaging (MRI), particularly using diffusion-weighted imaging (DWI) and apparent diffusion coefficient (ADC) sequences, is the most sensitive modality to diagnose brain injury following CA, with significantly-higher diagnostic value compared to computed tomography (CT). 37 Hyperintense areas on DWI reflect regions of decreased ADC, indicating restricted diffusion of water molecules across damaged cell membranes—an established consequence of cytotoxic edema secondary to HI brain injury. Additionally, the fluid-attenuated inversion recovery (FLAIR) sequence aids in detecting lesions near the ventricles. Magnetic resonance imaging findings showing diffuse abnormalities on DWI/ADC and FLAIR sequences provide strong evidence of poor neurological prognosis in this setting. 38

The timing of MRI acquisition is also critical. It is recommended 5 to perform the exam after 48 hours after ROSC, to avoid underestimating evolving injury processes that may not yet be radiologically evident.

A more affordable, faster, and widely-available alternative to MRI is CT. Although less sensitive to detect HI brain injury, 39 CT remains a useful and accessible tool to assess the extent of brain damage, aiding in neurological prognostication and therapeutic planning. 40 Moreover, CT enables the identification of treatable conditions such as intracranial hemorrhage or traumatic injuries that may significantly impact clinical management.

Two key time points are considered for CT in the context of post-CA assessment. First, an early CT scan, within the first 24 hours after the event, is recommended when the etiology of the CA is unclear, in cases of suspected traumatic brain injury (TBI), or when ROSC is delayed (> 20 minutes), and it may also inform decisions regarding therapies such as TTM. 41 Early CT reveals abnormalities in approximately 25% of the patients, most frequently associated with HI brain injury, 40 and such findings have been consistently linked 42 to poor outcomes (Cerebral Perfonance Category [CPC]. categories 4 or 5) and a higher performance of progression to brain death. However, the best sensitivity to predict poor outcomes is achieved when CT is performed between 48 and 96 hours post-CA, as this window offers improved detection of progressive HI damage. 43

Key CT findings suggestive of HI injury include: Loss of gray-white matter differentiation, effacement of cortical sulci, ventricular compression, all secondary to diffuse cerebral edema, typically appearing as generalized parenchymal hypodensity. 44 Other signs include obliteration of basal cisterns, due to severe cerebral edema and raised ICP, and hypodensities in deep brain structures such as the lentiform nucleus or thalamus, which are particularly vulnerable to hypoxia. 45 Additionally, the gray-to-white matter ratio (GWR) is a valuable quantitative parameter in detecting HI brain injury, with lower values being strongly associated with poor neurological outcomes. 43 46

### Prognostic classification of the patient

Based on sequential clinical evaluations and complementary diagnostic tests, patients can be classified into one of the stages of the CPC scale, which is widely used to standardize post-CA prognostication, with an emphasis on global neurological functionality, as described in Table 1 . 47 The CPC score is also used to predict long-term outcomes, with lower scores generally associated with higher survival rates and better quality of life. 48 Categories 4 and 5 of the CPC represent the most unfavorable prognoses, corresponding to persistent vegetative state (category 4) and brain death (category 5).

**Table 1:** Cerebral Performance Category (CPC) scale

Category	Outcome	Definition
1	Good recovery	Normal life or minimal neurological deficits
2	Moderate disability	Functional limitations, but able to live independently
3	Severe disability	Dependent on assistance for daily activities
4	Vegetative state	Without conscious interaction with the environment
5	Brain death	Complete absence of brain function

## DISCUSSION

The HCPA is a tertiary referral hospital serving the population of the state of Rio Grande do Sul, Brazil. The hospital is equipped with 860 beds, including 98 adult ICU beds. In 2024, HCPA recorded 67 cases of in-hospital CA. For patients who survive such events, neurological prognostication is commonly required. Based on the literature review herein conducted and considering the resources available at HCPA, we have designed an institutional protocol to standardize neurological assessment following CA.

For patients who survive CA, care begins immediately after ROSC in the intensive care setting. During the first 24 hours, it is recommended to initiate TTM, aiming for a core body temperature between 32 and 37.5 °C, especially in comatose patients. Continuous EEG monitoring should also be started. If continuous EEG is unavailable, a standard EEG may be performed to rule out SE, which would alter clinical management (a topic not addressed in this protocol). Non-contrast CT scans of the brain can be considered within the first 24 hours, particularly when the etiology of the arrest is unclear, if TBI is suspected, or if ROSC time exceeds 20 minutes. Supportive ICU care should be provided during this period, including continuous analgosedation as needed for patient comfort and to maintain hemodynamic stability, with a preference for short half-life agents.

The second day (25–48 hours postevent) is a maintenance phase aimed at clinical and neurological stabilization. Although NSE levels could be measured at this time, NSE testing is currently unavailable at HCPA and will not be included in this protocol. If TTM has been initiated, it may be maintained for up to 48 hours, or rewarming may begin if TTM cannot be sustained. Additionally, if the patient remains under continuous sedation, progressive weaning should be initiated, if feasible, to enable a reliable neurological examination in the coming days.

Only after 48 hours post-ROSC is it recommended to perform neuroimaging for prognostication purposes, preferably brain MRI. If MRI is unavailable, CT may be used. This delay ensures that radiological signs of brain injury are adequately developed. On day 3 (48–72 hours postevent), SSEPs—particularly analysis of the N20 wave—could be useful. However, due to technical limitations at HCPA, this test will not be incorporated into the institutional protocol.

On day 4, at least 72 hours after the event, clinical neurological examination becomes more reliable and should be performed in the absence of confounding factors, such as ongoing sedation, and with the patient in a clinically-stable state (with absence of electrolyte imbalances and controlled infections, for example). If TTM was not used, the neurological examination can be conducted at this point, with special attention to pupillary reflexes, other brainstem reflexes, and motor responses to painful stimuli. If TTM was applied, it is advisable to wait an additional 24 hours after rewarming to minimize the influence of temperature and sedation on the neurological assessment. During this period, a follow-up EEG may also be considered to detect ongoing seizure activity that could warrant specific treatment.

After approximately 120 hours post-CA and ROSC, further neurological reassessment and complementary testing should be individualized. Decisions regarding prognosis and therapeutic goals should be based on a multidisciplinary discussion involving neurology, intensive care, and other teams involved in patient care, in collaboration with the patient's family. Figure 1 provides a schematic flowchart summarizing recommended evaluations and interventions at each time point following ROSC.

**Figure 1:**
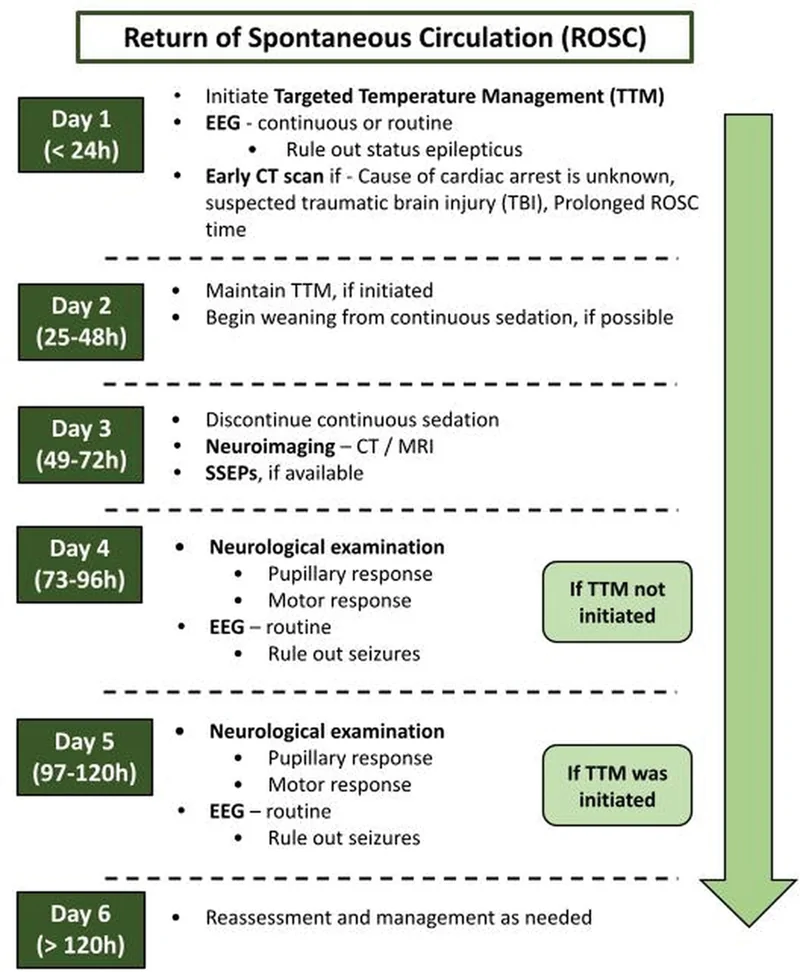
Neurological prognostication assessment flowchart after cardiac arrest. Adapted and modified from Rajajee et al., 16 2023.

A patient who is alert and consistently following commands with all four limbs suggests awakening from post-CA coma, indicating a higher likelihood of favorable neurological recovery. Motor responses such as withdrawal from or localization to painful stimuli are also considered favorable signs and are associated with good neurological outcomes at 3 months. In contrast, absent or extensor motor responses are not reliable predictors of poor outcome. On the other hand, when evaluating a patient in a non-perceptive comatose state, it is essential to promptly assess for criteria of brain death, and if appropriate, initiate the brain-death determination protocol according to HCPA guidelines.

Based on the combined clinical assessments by the neurology and ICU teams and the results of complementary exams, it is possible to estimate the probability of favorable or unfavorable outcomes. Figure 2 presents a flowchart illustrating likely neurological prognoses based on findings from each stage of the evaluation.

**Figure 2:**
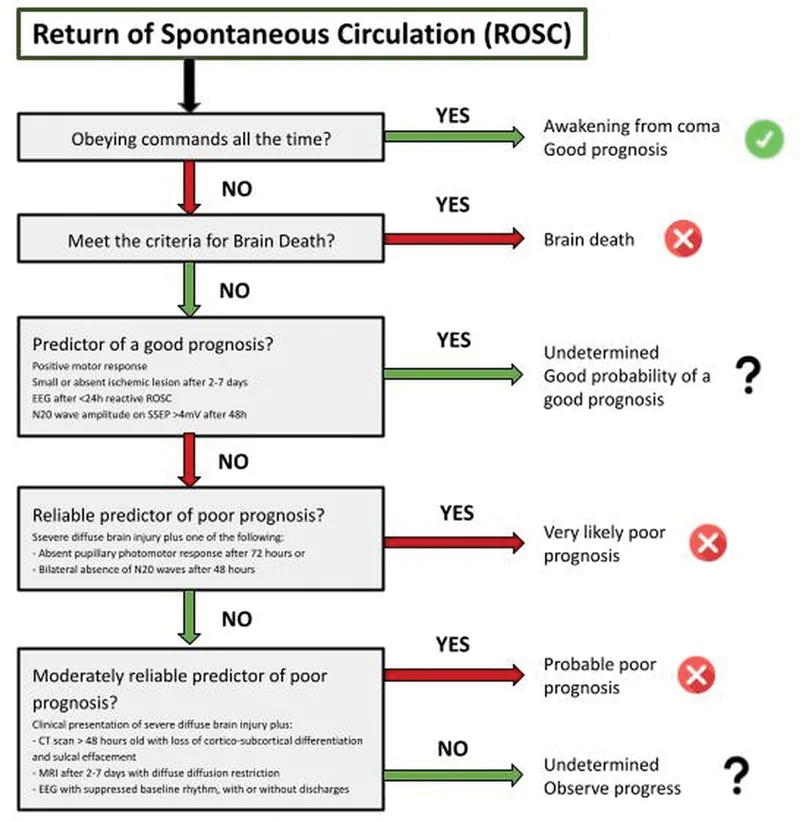
Flowchart of interpretation after post-cardiac arrest neurological evaluation. Adapted and modified from Rajajee et al., 16 2023.

Many patients follow a clinical course that results in neither awakening from coma nor progression to brain death. In these cases, detailed neurological assessment combined with ancillary testing becomes crucial to establish prognosis and to distinguish between potentially-favorable and unfavorable outcomes. 49

Numerous predictors of potentially-favorable neurological outcomes have been described, 8 provided they are assessed at the appropriate time and in the absence of confounding factors. These include: a positive motor response (limb withdrawal or localization of painful stimuli); neuroimaging, particularly MRI, showing either absent or small HI lesions on DWI performed between 2 to 7 days postevent; reactive EEG within 24 hours of ROSC; and bilateral presence of N20 waves with amplitude > 4 μV on SSEPs recorded after 48 hours post-ROSC. 8 The presence of one or more of these findings, although not necessarily all, suggests an indeterminate but potentially-favorable prognosis. In such cases, it is advisable to maintain life-sustaining therapies and allow for a prolonged observation period to assess for possible neurological recovery. 8

Nevertheless, CA is a severe event associated with high mortality rates. Consequently, many patients will lack favorable prognostic markers and ultimately progress to poor outcomes, such as death or persistent vegetative state. In this context, the multidisciplinary team must carefully assess strong predictors of poor neurological prognosis. The two most specific findings for a negative outcome are: Absence of bilateral pupillary light reflex at 72 hours post-ROSC (in the absence of ongoing sedation), and bilateral absence of N20 waves on SSEPs performed after 48 hours. These findings, whether observed in isolation or in combination (and particularly in the presence of clinical suspicion of severe diffuse brain injury) are highly predictive of irreversible poor neurological outcomes.

Some patients may remain comatose after CA without presenting the most specific predictors of poor prognosis. In such cases, additional markers must be considered to estimate the likely trajectory. Neuroimaging plays a key role here by identifying diffuse HI brain injury, which serves as a moderately-reliable predictor of unfavorable outcomes. Moreover, the presence of an EEG showing a suppressed background pattern, with or without epileptiform discharges, also suggests a poor prognosis. If, after serial clinical examinations and complementary testing, no predictors of poor neurological outcome are present, it is recommended to maintain supportive care and continue close observation. This approach enables the identification of signs of progressive neurological recovery and helps prevent premature decisions regarding the withdrawal of life-sustaining treatment.

Finally, to standardize neurological-outcome assessment in post-CA patients and provide an objective and global measure of neurological function, it is recommended to classify the patient according to the CPC scale ( Table 1 ), which includes five categories. This classification facilitates long-term outcome prediction, improves communication between healthcare teams and families, and supports therapeutic decision-making and evaluation of clinical interventions. A systematic approach such as this promotes more structured and evidence-based care, aligns expectations with families, and optimizes treatment strategies based on each patient's condition and potential for recovery.

In conclusion, CA is a critical event associated with high rates of mortality and neurological morbidity. Appropriate management and neuroprognostication—based on clinical and ancillary tools—are essential to optimize care and guide therapeutic decision-making. The current study reviewed the main evidence regarding neurological outcomes after CA and proposed an institutional protocol tailored to the reality and available resources at HCPA.

The implementation of a standardized protocol for neurological assessment has the potential to improve the quality of care and to enhance decision-making confidence, reduce uncertainty, and align expectations with patients' families. Ultimately, this approach aims to provide more ethical, humanized, and evidence-based care for patients recovering from CA.
